# Serum KL-6 combined with immune/inflammatory biomarkers identifies complicated silicosis

**DOI:** 10.1186/s12890-025-04091-0

**Published:** 2026-01-03

**Authors:** Rui He, Limin Huang, Yang Chen, Minqi Liu, Miaomiao Xie, Honglei Yuan, Ling Mao

**Affiliations:** Department of Pneumoconiosis, Shanghai Pulmonary Hospital, Tongji University School of Medicine, 507 Zhengmin Road, Shanghai, 200433 China

**Keywords:** ESR, KL-6, LDH, Pulmonary function, Serum biomarkers, Silicosis

## Abstract

**Background:**

Complicated silicosis (CS) poses significant diagnostic challenges, with current diagnosis relying heavily on radiographic imaging. These challenges highlight a need for reliable non-invasive biomarkers to improve early detection and disease staging. This study aimed to evaluate serum Krebs von den Lungen-6 (KL-6) levels in silicosis patients across different disease stages and explore their association with immune-inflammatory markers to assess its potential as a biomarker for early diagnosis and risk stratification.

**Methods:**

This study enrolled individuals evaluated at our institution between October 2022 and June 2025 and categorized them into three groups: dust-exposed workers without silicosis (DEWs), patients with simple silicosis (SS), and those with CS. Serum levels of KL-6, interleukin (IL)-6, IL-8, IL-10, CD8^+^ T cells, and other inflammatory markers were measured, and the erythrocyte sedimentation rate (ESR) was assessed alongside pulmonary function tests. Receiver operating characteristic (ROC) curves and correlation analyses were used to assess diagnostic performance and biomarker associations.

**Results:**

In total, 135 individuals were enrolled in this study. Of these, 25 were categorized as DEWs, 33 as patients with SS, and 77 with CS. Patients with CS were younger than those with SS (median age: 59.0 vs. 68.0 years) and had a shorter exposure duration (mean: 16 vs. 18 years) but worse lung function. KL-6 levels increased progressively across groups, peaking in the CS group (SS vs. CS: median, 257.00 vs. 441.22 U/mL; IQR: 202.96–358.50 vs. 295.53–926.50 U/mL). KL-6 was negatively correlated with FVC%, FEV_1_%, and DLCO% and positively correlated with lactate dehydrogenase (LDH) (*r* = 0.57) and ESR (*r* = 0.44). At a cutoff of 300 U/mL, KL-6 distinguished CS from SS with an area under the curve (AUC) of 0.756 (sensitivity, 75.3%; specificity, 69.7%) and performed better in distinguishing CS from DEWs (AUC = 0.842; sensitivity, 74%; specificity, 84%). A combined model of KL-6, LDH, and ESR further improved diagnostic accuracy (AUC = 0.914; sensitivity, 85.7%; specificity, 80.0%).

**Conclusions:**

Serum KL-6 is strongly associated with pulmonary function decline and systemic inflammation in silicosis. When combined with LDH and ESR, it significantly enhances diagnostic precision, offering a promising non-radiological biomarker panel for disease staging and early identification of disease severity.

## Background

Silicosis is a progressive fibrotic lung disease caused by prolonged occupational inhalation of crystalline silica (SiO₂) [1]. Even after exposure ceases, retained silica particles trigger chronic inflammation and lung tissue injury, resulting in irreversible fibrosis [2]. Radiographically, silicosis is classified as simple or complicated. Complicated silicosis (CS), or progressive massive fibrosis (PMF), is characterized by coalescent nodules over 1 cm in size and extensive lung damage. It is common in high-risk occupations, such as stone cutting and sandblasting, and is associated with younger age of onset, shorter exposure duration, rapid progression, and higher comorbidity and mortality rates [3, 4]. In China, silicosis remains the most prevalent occupational disease, representing approximately 79% of pneumoconiosis cases by 2021 [5], and continues to pose a significant public health burden in developing countries.

Current diagnostic methods for silicosis include chest radiography, high-resolution computed tomography (HRCT), pulmonary function tests (PFTs), and the 6-minute walk test (6MWT). While clinically useful, these tools have limitations regarding sensitivity and applicability. PFTs require patient cooperation and may be unreliable in cases of pneumothorax, emphysema, or respiratory failure [6], whereas the 6MWT is affected by musculoskeletal or cardiovascular conditions. HRCT provides detailed imaging, but repeated use increases radiation exposure, limiting its suitability for long-term monitoring [7]. Importantly, both imaging and functional tests often detect disease only after significant structural damage has occurred, hindering early diagnosis, which is particularly critical in rapidly progressing CS [8]. Therefore, non-invasive, radiation-free, and reliable serum biomarkers are urgently needed to enable earlier disease detection, track progression, and improve clinical decision-making.

Krebs von den Lungen-6 (KL-6), a mucin-like glycoprotein secreted by alveolar type II and bronchial epithelial cells [9, 10], has emerged as a promising biomarker in interstitial lung diseases, chronic obstructive pulmonary disease, and COVID-19-related pneumonia [11–13]. KL-6 is closely associated with epithelial injury and disease severity in fibrotic lung disorders [14]. Although elevated KL-6 levels have been reported in silicosis, data on its role across disease stages remain limited, particularly in Chinese cohorts. Additionally, inflammatory cytokines, such as interleukin (IL)-6, IL-8, IL-10, and CD8^+^ T cells, play critical roles in modulating immune responses [15–17]. Their interaction with KL-6 may have diagnostic and prognostic relevance, yet their association remains underexplored.

This study aimed to evaluate serum KL-6 levels in patients with silicosis across different disease stages and explore their association with immune-inflammatory markers to assess the former’s potential as a biomarker for early diagnosis and risk stratification.

## Methods

### Patient characteristics

This was a study based on available samples. Clinical data were collected from 485 patients who were hospitalized with occupational dust exposure at the Department of Pneumoconiosis of our research hospital, and from 25 dust-exposed individuals who visited for routine occupational disease screening, between October 2022 and June 2025. All eligible patients were consecutively enrolled, and none had a prior history of malignancy or occupational radiation exposure. The exclusion criteria included: (1) incomplete clinical or laboratory data, (2) concurrent respiratory diseases (e.g., tuberculosis, lung cancer) or acute exacerbations triggered by acute respiratory infections, and (3) prior use of antifibrotic medications. In accordance with these criteria, we excluded 35 patients with active pulmonary tuberculosis, 16 with lung cancer, 196 hospitalized for acute pulmonary infection, 86 with incomplete clinical records, and 42 with a history of antifibrotic therapy. In the final cohort, 25 participants were classified as dust-exposed workers without silicosis (DEWs), and 110 were diagnosed with silicosis.

**Fig. 1:**
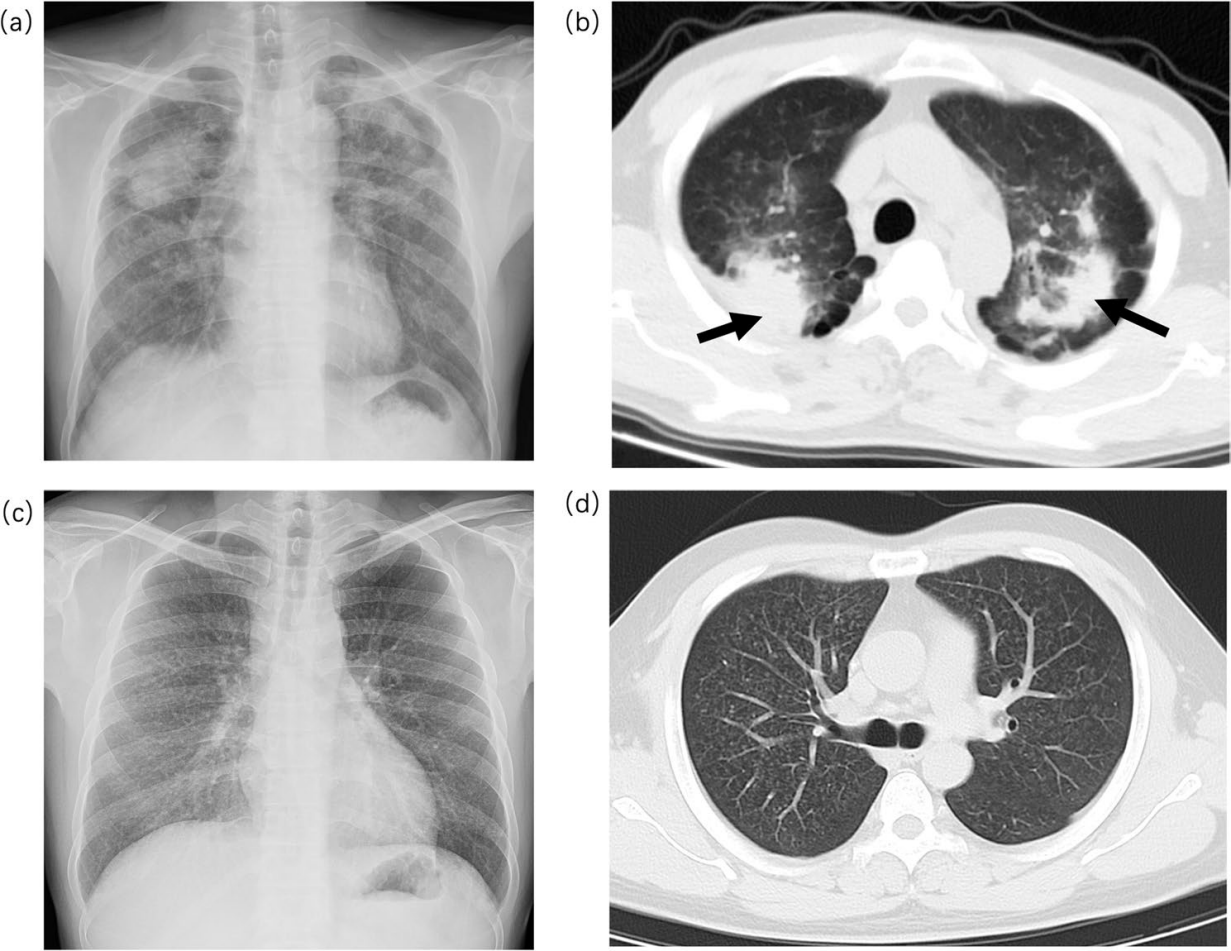
Typical imaging features of simple silicosis (CS) and complicated silicosis (SS). **a**, **b** Representative chest radiograph and high-resolution computed tomography images of CS, with the arrow indicating a progressive massive fibrosis lesion. **c**, **d** Representative chest radiograph and high-resolution computed tomography images of SS

The patients were diagnosed with silicosis according to the diagnostic criteria for pneumoconiosis in China, with a chest radiograph showing profusion category 1 opacities involving at least two lung zones serving as the diagnostic threshold. Differentiation between simple silicosis (SS) and CS was primarily based on chest radiographs, supplemented by HRCT findings (Fig. 1). SS is characterized by diffusely distributed small nodules (1–10 mm), whereas CS exhibits a mass > 1 cm consistent with PMF [18]. DEWs showed no radiological signs of silicosis. All diagnoses were made by a specialized occupational disease diagnostic team comprising senior physicians certified in pneumoconiosis diagnosis and were subsequently reviewed by the hospital’s senior diagnostic committee. Radiologists were blinded to all clinical information and biomarker results except for dust-exposure history. Serum levels of KL-6, IL-6, IL-8, IL-10, CD8⁺ cells, creatine kinase (CK), and lactate dehydrogenase (LDH) were measured. In addition, erythrocyte sedimentation rate (ESR), white blood cell count (WBC), lymphocyte count (LYM), neutrophil count (NEUT), pulmonary function parameters, and demographic data (age, sex, smoking history, exposure duration) were recorded.

### Serum biomarker measurement

Venous blood samples (5 mL) were collected between 6:00 and 8:00 a.m. after overnight fasting and stored at − 80° C until analysis. Serum KL-6 was measured using a chemiluminescent immunoassay (Fujirebio, Japan; detection limit 10–25 U/mL). IL-6, IL-8, IL-10, tumor necrosis factor-α, and N-terminal pro-B-type natriuretic peptide (NT-proBNP) were quantified on the Roche Cobas e platform (Switzerland), with detection limits of approximately 0.5-3 pg/mL for cytokines and < 5 pg/mL for NT-proBNP. T-lymphocyte subsets and hematologic parameters were analyzed using the Beckman Coulter DxFLEX flow cytometer and Abbott CELL-DYN analyzer, respectively. CK and LDH were measured on the Roche Cobas C system; CK was primarily measured using the enzyme-coupled rate method, and LDH was assayed via the L-P enzyme method. ESR was assessed using the Westergren method (Sysmex TEST1). All assays followed routine internal and external quality-control procedures. Samples were processed within recommended time windows and were not subjected to repeated freeze-thaw cycles.

### PFTs

Pulmonary function was assessed using the Jaeger MasterScreen system (Germany). PFTs were performed within 72 h of blood collection and included forced vital capacity (FVC), forced expiratory volume in 1 s (FEV_1_), FEV_1_/FVC ratio, and diffusing capacity of the lung for carbon monoxide (DLCO). Each test was repeated at least three times, and the best-effort curve that met the ATS/ERS quality criteria was selected for analysis. DLCO values were corrected for hemoglobin concentration. Considering that all participants were clinically stable and imaging examinations (including HRCT) were routinely performed on the day of admission to rule out contraindications such as pneumothorax, PFTs were typically conducted starting on the following day.

### Statistical analysis

Data normality was assessed using one-way analysis of variance (ANOVA). Continuous variables were analyzed using independent-sample t-tests or Mann-Whitney U tests, as appropriate, and expressed as mean ± SD or median (P25, P75). Missing values for a small number of variables were handled using multiple imputation in SPSS (Version 23.0, IBM Corp., Armonk, NY, USA). Spearman’s correlation was used to assess associations between variables. Receiver operating characteristic (ROC) curves and the Youden index were applied to evaluate diagnostic performance and optimal cut-off values of the biomarkers. The DeLong test was used to further compare the reliability of areas under the curve (AUCs) between groups. Correlation heatmaps were generated to visualize relationships among biomarkers, and variables showing the strongest correlations were subsequently incorporated into logistic regression models for combined diagnostic analysis. Logistic regression was used to assess combined diagnostic value and compare model performance across groups. Statistical analyses were conducted using SPSS 23.0 and GraphPad Prism 10.4, with *P* < 0.05 considered significant.

## Results

### Demographic and clinical characteristics

A total of 135 participants were enrolled, including 77 individuals with CS, 33 with SS, and 25 DEWs. CS participants were significantly younger than SS participants (median, 59.0 [IQR: 50.0–66.0] vs. 68.0 [56.5–76.0] years; *P* < 0.001), had a shorter dust exposure duration (16.53 ± 10.61 vs. 18.79 ± 10.41 years; *P* = 0.060), and exhibited greater pulmonary function impairment. Significant intergroup differences were observed in LYM, CD8^+^ T cells, CD4^+^/CD8^+^ ratio, IL-10, ESR, LDH, CK, and KL-6 levels (Table 1). Notably, KL-6 levels increased progressively across the DEWs, SS, and CS groups, with the highest median observed in patients with CS (median, 441.22 [IQR: 295.53–926.50] U/mL), indicating a strong association with disease severity. Pulmonary function indices, including FVC% (71.79 ± 25.63), FEV_1_% (57.92 ± 28.55), and DLCO% (81.75 ± 38.00), were significantly lower in CS than in SS and DEWs (all *P* < 0.001), highlighting substantial functional decline in advanced disease.

**Table 1:** Baseline characteristics of the patients

Groups	DEWs	SS	CS	*P*-value
N	25	33	77	
Male: Female	25:0	32:1	77:0	0.213
Age (years)	55.0 (52.0–59.0)	68.0 (56.5–76.0)	59.0 (50.0–66.0)	< 0.001
Dust exposure time (years)	12.38 ± 8.23	18.79 ± 10.41	16.53 ± 10.61	0.060
Smoker: Non- smoker*	19:06 (76%)	15:18 (45%)	48:29 (62%)	0.057
smoking index (pack/year)	21.12 ± 18.72	12.88 ± 19.46	19.90 ± 26.65	0.302
Monocyte Count (×10^9^/L)	0.54 ± 0.14	0.56 ± 0.19	0.58 ± 0.24	0.668
WBC (×10^9^/L)	6.78 ± 1.90	6.48 ± 2.14	6.91 ± 2.58	0.687
LYM (×10^9^/L)	1.47(1.08–2.34)	1.59(1.34–2.01)	1.28(0.87–1.60)	< 0.001
NEUT (×10^9^/L)	4.24 ± 1.43	4.00 ± 1.85	4.86 ± 2.32	0.106
CD4^+^	41.80(28.74–50.37)	41.87(35.80-46.63)	37.11(31.70–43.70)	0.225
CD8^+^	21.92 ± 9.89	18.56 ± 6.34	24.12 ± 9.05	0.010
CD4^+^/CD8^+^	2.25 ± 1.36	1.61 ± 1.55	1.81 ± 0.90	0.005
IL6 (pg/mL)	5.72(3.83–15.74)	5.77(2.70-11.07)	7.57(3.30-14.61)	0.415
IL8 (pg/mL)	28.09 ± 30.24	33.79 ± 23.01	40.64 ± 26.19	0.094
IL10 (pg/mL)	3.70 ± 1.97	4.54 ± 3.33	5.25 ± 2.72	0.047
TNF (pg/mL)	1.49(0.21–2.69)	1.04(0.62–2.15)	1.21(0.63–2.06)	0.960
ESR (mm/H)	21.72 ± 26.31	29.46 ± 32.54	54.82 ± 32.92	< 0.001
LDH (IU/L)	159.00 (128.85-175.35)	187.50 (153.10-216.75)	206.90 (178.25–271.80)	< 0.001
CK (IU/L)	65.00(52.30–77.20)	79.60(61.75-98.00)	52.80(41.00–80.00)	0.001
KL6 (U/mL)	240.00 (156.98-291.43)	257.00 (202.96–358.50)	441.22 (295.53–926.50)	< 0.001
NT-proBNP (pg/mL)	113.00 ± 186.47	278.21 ± 921.93	313.35 ± 851.16	0.547
Pulmonary function				
FVC (L)	3.40 ± 1.00	2.99 ± 0.81	3.12 ± 5.54	0.936
FVC, %pred	99.08 ± 21.37	95.10 ± 27.08	71.79 ± 25.63	< 0.001
FEV_1_, %pred	90.82 ± 23.85	87.85 ± 30.94	57.92 ± 28.55	< 0.001
FEV_1_/FVC (%)	89.20(78.50–99.90)	76.18(68.12–96.05)	84.10(62.65–99.20)	0.177
DLCO, %pred	113.00 ± 32.07	103.93 ± 42.72	81.75 ± 38.00	< 0.001

Intergroup comparisons of KL-6 and immune-inflammatory markers were performed between the CS and DEWs groups, as well as between the CS and SS groups. Prior to analysis, homogeneity of variance and data normality were assessed. For variables following a normal distribution, one-way ANOVA was used to evaluate statistical significance. For non-normally distributed variables, non-parametric tests were applied

*Smoking population: refers to individuals with a history of smoking, including both former smokers (individuals who previously smoked but have quit) and current smokers. The percentage indicates the proportion of smokers within each group

*CS* Complicated silicosis, *SS* Simple silicosis, *DEWs* Dust-exposed workers without silicosis, *CD4*^*+*^Cluster of differentiation 4 positive T cells, *CD8*^*+*^Cluster of differentiation 8 positive T cells, *CD4+/CD8+*  CD4 to CD8 T-cell ratio, *CK* Creatine kinase, *DLCO* % pred, diffusing capacity of the lung for carbon monoxide, percent of predicted value, *ESR* Erythrocyte sedimentation rate, *FEV1/FVC(%)* Forced expiratory volume in 1 second to forced vital capacity ratio, *FEV1* %pred, forced expiratory volume in 1 second, percent of predicted value, *FVC* % pred, forced vital capacity, percent of predicted value, *FVC(L)* Forced vital capacity in liters, *IL6* Interleukin-6, *IL8* Interleukin-8, *IL10* Interleukin-10, *KL-6* Krebs von den Lungen-6, *LDH* Lactate dehydrogenase, *LYM* Lymphocyte count, *NEUT* Neutrophil count, *NT-pro BNP* N-terminal pro-B-type natriuretic peptide, *TNF* Tumor necrosis factor, *WBC* White blood cell count, ×*10*^9^/*L* units: 10 to the power of 9 per liter,  *pg/mL* units: picograms per milliliter,* IU/L* units: international units per liter, *mm/H* units: millimeters per hour, *U/mL* units: units per milliliter

### KL-6 and Immune/Inflammatory marker expression

**Fig. 2:**
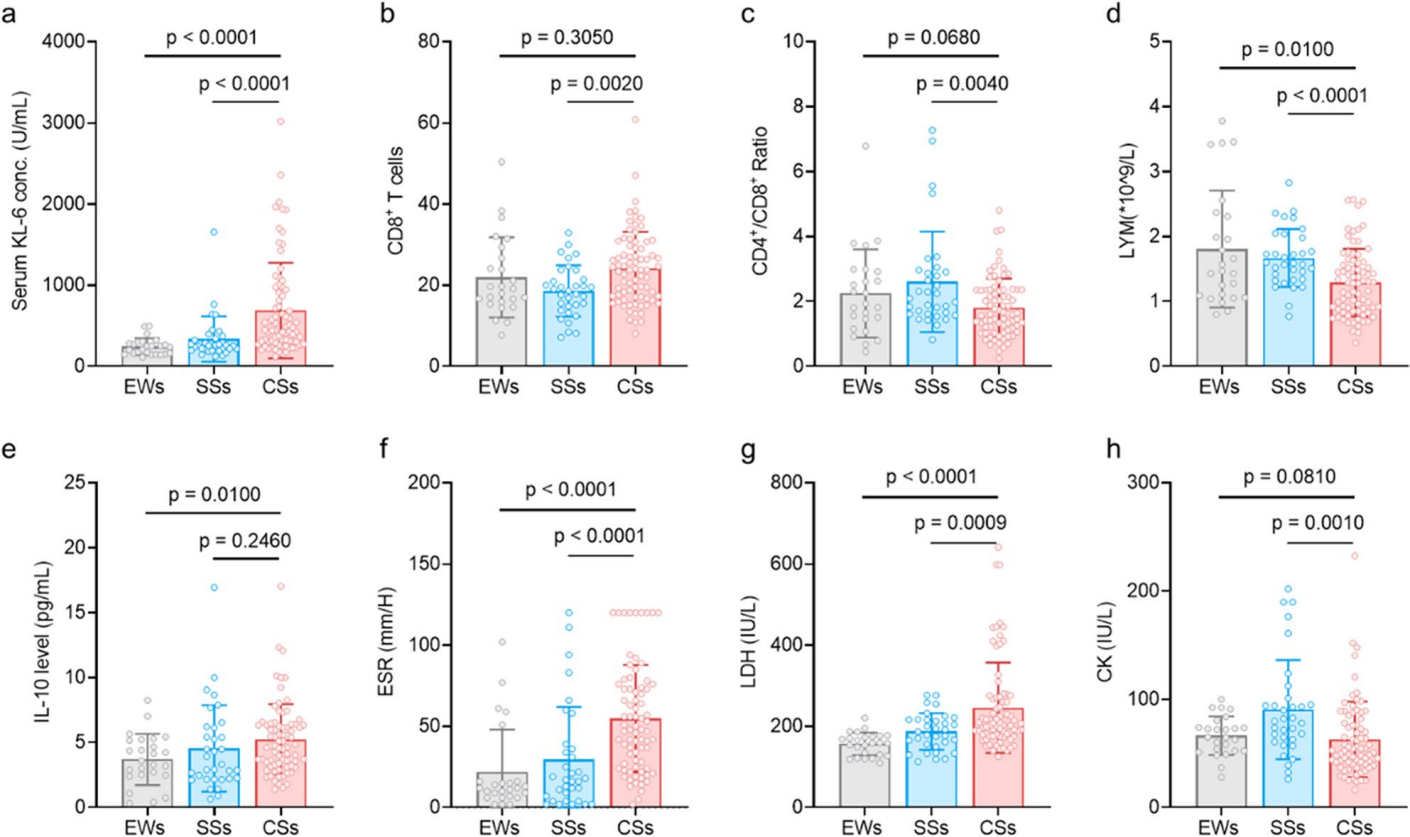
Expression of serum Krebs von den Lungen-6 (KL-6) and different immune/inflammatory markers in the dust-exposed individuals without silicosis (DEWs), SS, and CS groups. *P* < 0.05 indicates statistically significant differences. **a**-**h** Comparisons of serum KL-6, CD8+ T-cell, CD4+/CD8+ ratio, lymphocyte count, IL-10 level, ESR, LDH level, and CK level between CS group and DEWs group/SS group, respectively

Based on the statistically significant variables shown in Table 1, we analyzed serum levels of KL-6 and selected immune/inflammatory markers in the DEWs, SS, and CS groups (Fig. 2). KL-6, LYM, LDH, and ESR were significantly higher in the CS group than in the SS and DEWs groups, showing an upward trend with disease severity and effectively distinguishing CS. CD8^+^ T cells were significantly increased in the CS group compared with those in the SS group (*P* = 0.0020), but not significantly different between the CS and DEWs groups (*P* = 0.3050), limiting their discriminative power between exposed individuals and those with advanced disease. IL-10 levels were also elevated in the CS group compared with those in the DEWs group (*P* = 0.0100), although not significantly different from those in the SS group (*P* = 0.2460). These findings suggest that KL-6, LYM, and inflammatory markers (LDH and ESR) are sensitive markers for identifying severe silicosis, whereas CD8^+^ T cells are more useful for distinguishing between SS and CS.

### KL-6 correlates with pulmonary function

**Fig. 3:**
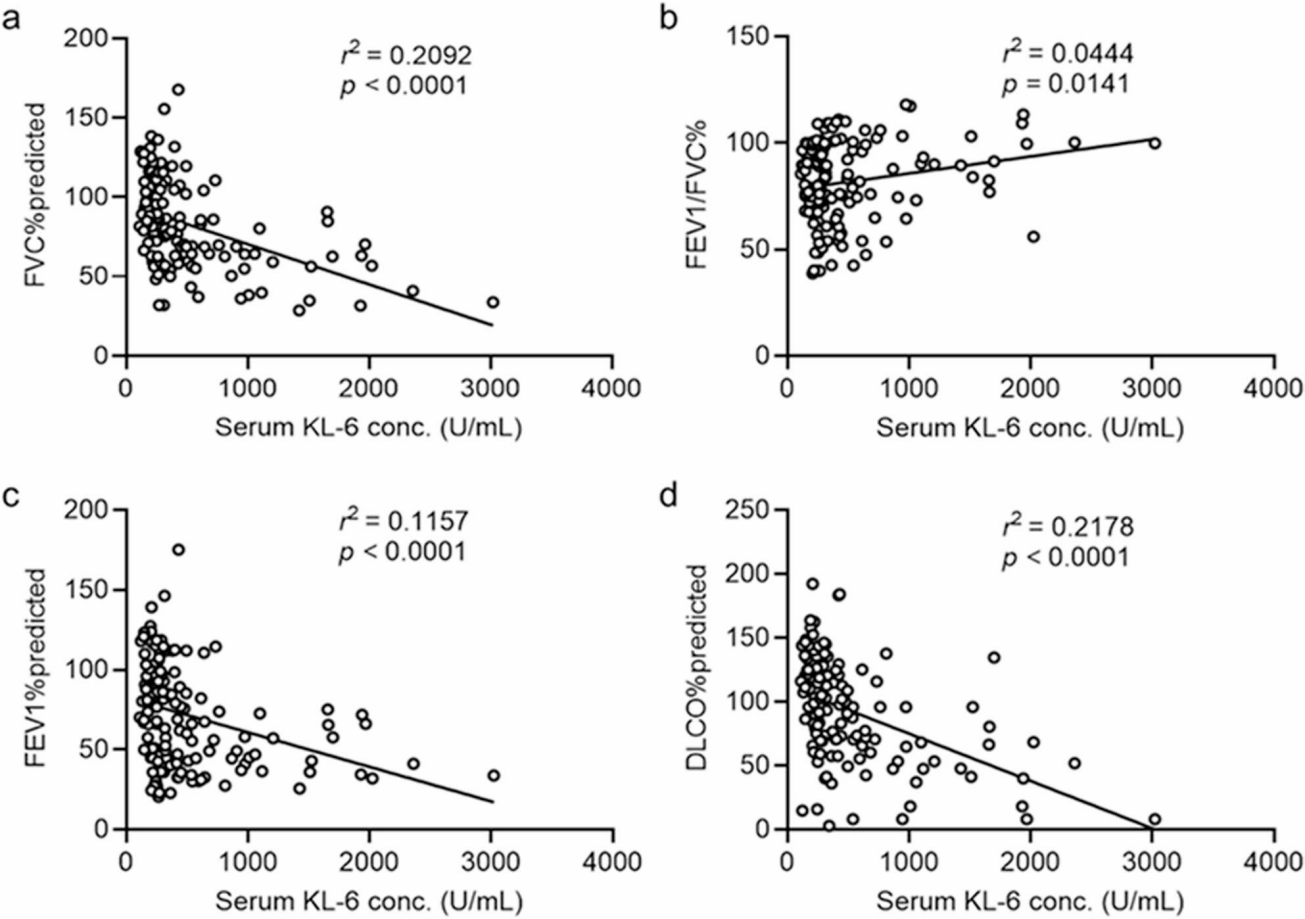
Correlations between serum KL-6 levels and pulmonary function parameters. **a**-**d** Correlations of KL-6 with FVC% predicted, FEV_1_/FVC%, FEV_1_% predicted, and DLCO% predicted. Statistical significance was set at *P* < 0.05

To further explore the clinical significance of KL-6, the relationship between KL-6 and key indicators of pulmonary function was assessed. As shown in Fig. 3, serum KL-6 levels were significantly and negatively correlated with FVC% predicted (*P* < 0.0001), FEV_1_% predicted (*P* < 0.0001), and DLCO% predicted (*P* < 0.0001), suggesting that elevated KL-6 levels reflect a greater impairment in ventilatory capacity and gas exchange. KL-6 levels were positively correlated with FEV_1_/FVC% (*P* = 0.0141).

### KL-6 ROC curve analysis

**Fig. 4:**
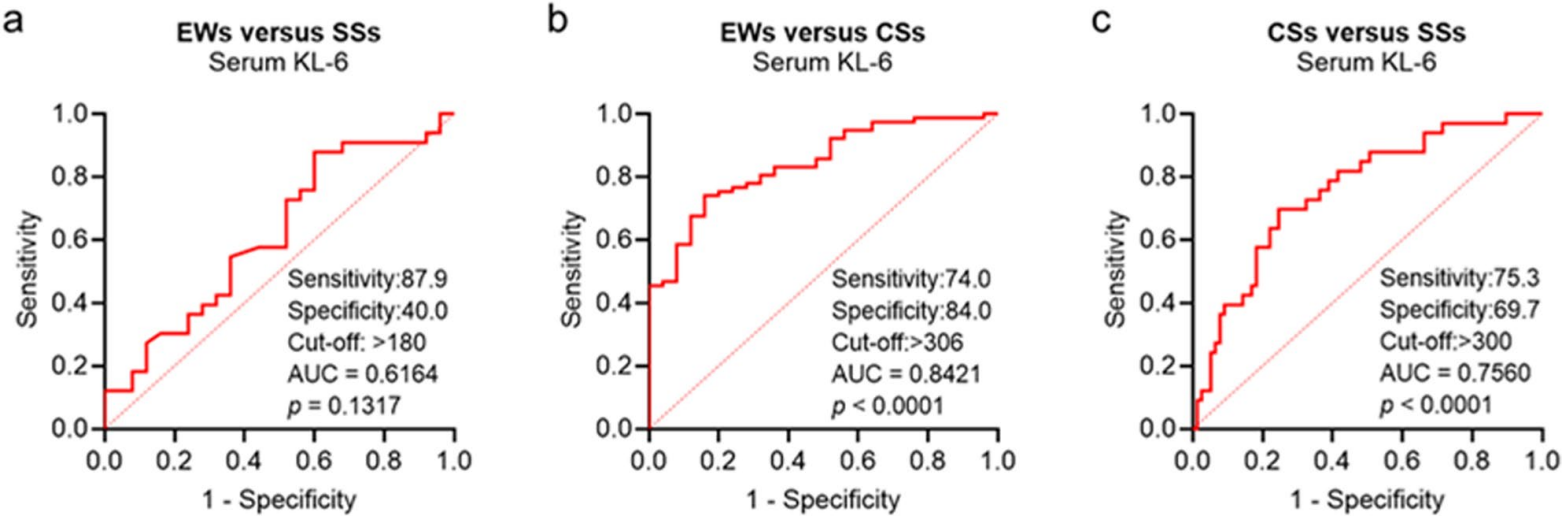
Receiver operating characteristic curve analysis of serum KL-6 in different comparison groups. **a** Diagnostic performance between DEWs and SS, (**b**) between DEWs and CS, and (**c**) between SS and CS. *P* < 0.05 indicates statistical significance

To assess the diagnostic performance of serum KL-6 across disease stages, we performed ROC curve analyses comparing the DEWs, SS, and CS groups (Fig. 4). KL-6 demonstrated the highest diagnostic accuracy in distinguishing CS from DEWs, with an AUC of 0.842, sensitivity of 74%, specificity of 84%, and an optimal cutoff of 306 U/mL (*P* < 0.0001, 95% CI: 0.762–0.922). While its ability to differentiate SS from DEWs or CS was moderate (AUC = 0.616 and 0.756, respectively), KL-6 showed the greatest potential for identifying advanced disease among exposed individuals, underscoring its clinical value for the early detection of severe silicosis.

### KL-6 correlates with inflammatory markers

**Fig. 5:**
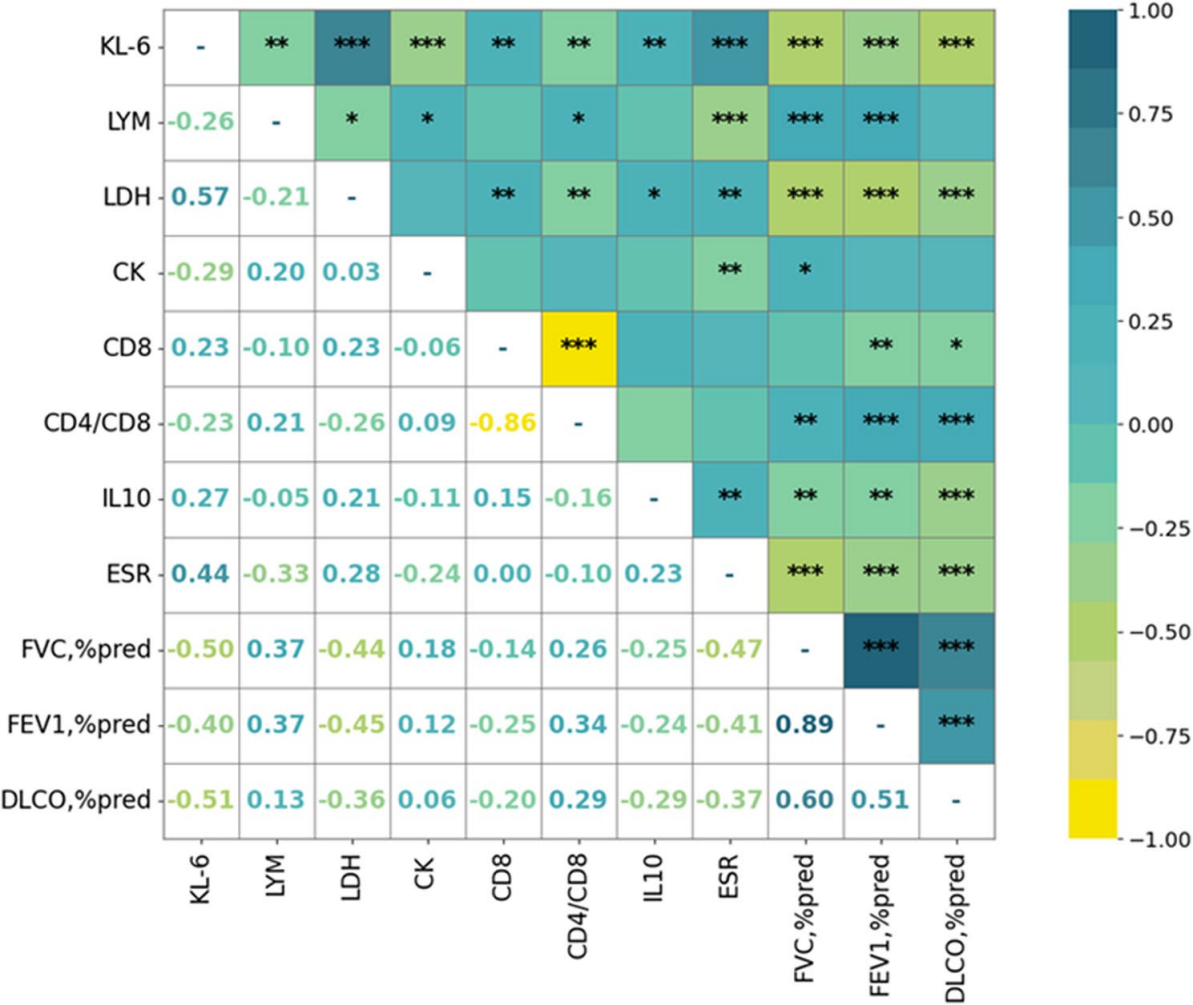
Heatmap showing Pearson correlation coefficients between serum KL-6 and immune, inflammatory, and pulmonary function parameters. Positive correlations are marked in green/blue and negative in yellow. Stronger correlations are indicated by coefficients closer to ± 1. *P* < 0.05 indicates statistical significance

To further explore the clinical relevance of KL-6, we constructed a correlation heatmap to examine associations with inflammatory markers and lung function parameters (Fig. 5). KL-6 levels were positively correlated with LDH (*r* = 0.57), CD8^+^ T cells, IL-10, and ESR, and negatively correlated with LYM, CK, CD4^+^/CD8^+^ ratio, and all key pulmonary function indicators (FVC% predicted, FEV_1_% predicted, and DLCO% predicted). Notably, the strongest associations were observed between KL-6 and LDH (*r* = 0.57), and between KL-6 and ESR (*r* = 0.44), suggesting that elevated KL-6 levels reflect both systemic inflammation and impaired pulmonary function in silicosis.

### Combined model performance

To evaluate the diagnostic value of combining KL-6 with inflammatory and immune markers, binary logistic regression models were constructed, and ROC analyses were performed to assess their ability to distinguish CS from other groups. To differentiate CS from DEWs, serum KL-6 alone achieved an AUC of 0.842 (sensitivity, 74.0%; specificity, 84.0%). Model performance improved when KL-6 was combined with LDH (AUC = 0.868) or ESR (AUC = 0.891). The full model (KL-6 + LDH + ESR) yielded the highest accuracy (AUC = 0.914, sensitivity: 85.7%, specificity: 80.0%), demonstrating enhanced diagnostic power through biomarker integration (Table 2).

**Table 2:** Diagnostic performance of individual and combined serum biomarkers (KL-6, LDH, ESR) for distinguishing complicated silicosis (CS) from dust-exposed workers without silicosis (DEWs)

Indicator	AUC	*P*-value	Sensitivity	Specificity	95%CI
KL-6	0.842	< 0.0001	74.0%	84.0%	0.762–0.922
KL-6 + LDH	0.868	< 0.0001	68.8%	92.0%	0.797–0.939
KL-6 + ESR	0.891	< 0.0001	90.9%	80.0%	0.814–0.969
KL-6 + LDH + ESR	0.914	< 0.0001	85.7%	80.0%	0.853–0.975

*AUC* Area under the ROC curve, *CI* Confidence interval, *KL-6* Krebs von den Lungen-6, *LDH* Lactate dehydrogenase, *ESR* Erythrocyte sedimentation rate

For CS vs. SS, KL-6 alone had an AUC of 0.756. The addition of LDH increased specificity (87.9%) but reduced sensitivity (50.6%). The KL-6 + ESR model offered a more balanced performance (AUC = 0.891), while the full model (KL-6 + LDH + ESR) reached an AUC of 0.801, with improved sensitivity and specificity (75.3% and 78.8%, respectively; Table 3). These findings suggest that integrating KL-6 with LDH and ESR improves diagnostic accuracy, particularly in identifying individuals with advanced silicosis.

**Table 3:** Diagnostic performance of individual and combined serum biomarkers (KL-6, LDH, ESR) for distinguishing complicated silicosis (CS) from simple silicosis (SS)

Indicator	AUC	*P*-value	Sensitivity	Specificity	95%CI
KL-6	0.756	< 0.0001	75.3%	69.7%	0.659–0.853
KL-6 + LDH	0.868	< 0.0001	50.6%	87.9%	0.636–0.835
KL-6 + ESR	0.891	< 0.0001	70.1%	78.8%	0.700–0.889
KL-6 + LDH + ESR	0.801	< 0.0001	75.3%	78.8%	0.711–0.891

*AUC* Area under the ROC curve, *CI* Confidence interval, *KL-6* Krebs von den Lungen-6, *LDH* Lactate dehydrogenase, *ESR* Erythrocyte sedimentation rate

## Discussion

Silicosis is an irreversible occupational fibrotic lung disease characterized by high progression and disability rates [19]. Compared with SS, CS presents with faster clinical deterioration and poorer prognosis [16]. Currently, diagnosis relies primarily on radiological imaging [20], while research on serum biomarkers remains limited and largely unsystematic despite recent advances [21–24]. In this study, patients were stratified into three groups—DEWs, SS, and CS—to comprehensively compare KL-6 levels and key immune-related indicators, aiming to identify a serum-based biomarker panel for early recognition of advanced disease.

KL-6 is a high-molecular-weight glycoprotein produced by alveolar type II and bronchial epithelial cells. Elevated KL-6 levels reflect alveolar epithelial injury and repair and are recognized as biomarkers of interstitial lung diseases and pneumoconiosis [21, 25]. In our study, KL-6 levels were significantly higher in the CS group than in the SS and DEWs groups. ROC analysis showed that a KL-6 threshold of 306 U/mL yielded an AUC of 0.842 and 84% specificity for diagnosing CS, indicating strong standalone diagnostic performance and utility in disease stratification.

We found that patients with higher KL-6 levels had relatively poorer pulmonary function indicators. These findings underscore the clinical potential of KL-6 not only as a disease severity marker but also as a surrogate for functional decline in silicosis, particularly in CS where early detection is critical for timely intervention.

Beyond its role as an epithelial injury marker, our study revealed a potential association between KL-6 and T cell subsets. CD8^+^ T cell expression was markedly elevated in the CS group but downregulated in the SS group, whereas the CD4^+^/CD8^+^ ratio was significantly reduced in the SS group, suggesting immune cell remodeling during disease progression. This pattern does not indicate a negative association between CD8⁺ T cells and disease severity but rather reflects the stage-specific immunological shifts in silicosis. In the early SS stage, silica-induced macrophage dysfunction promotes the release of immunosuppressive mediators such as IL-10 and transforming growth factor-β, creating a “low-activation, high-suppression” milieu that dampens both CD4⁺ and CD8⁺ T-cell responses [26]. With progression to CS, persistent epithelial injury and fibrosis lead to continuous release of damage-associated molecular patterns, which recruit and activate cytotoxic CD8⁺ T cells, resulting in their increased levels in advanced disease [27]– [28]. This shift from early immunosuppression to later cytotoxic activation represents the immune remodeling accompanying silicosis progression. Given this immune status and concurrent KL-6 elevation, KL-6 may also reflect disease-related immune activation in CS. Although IL-6 and IL-8 levels did not differ significantly across groups, IL-10 was significantly upregulated in CS, consistent with previous studies, suggesting roles in both chronic inflammation and tissue repair [24].

Notably, LDH and ESR (conventional inflammatory markers) reflect tissue injury and systemic inflammation when elevated [29, 30]. Our findings revealed a significant positive correlation between KL-6 and both LDH and ESR, indicating potential synchronous alterations during disease progression. Combining KL-6 with LDH enhanced diagnostic specificity, while pairing KL-6 with ESR improved sensitivity. The combination of KL-6, LDH, and ESR achieved the highest diagnostic performance (AUC = 0.914), substantially exceeding that of any single marker, offering a practical tool for serum-based screening of CS.

Beyond their diagnostic utility, although the present study primarily demonstrates the diagnostic value of KL-6 and related inflammatory markers in reflecting current disease severity, these biomarkers may also have potential prospective implications. KL-6 is produced by injured alveolar epithelial cells and has been shown to increase during early epithelial damage prior to overt radiological fibrosis [9, 10]. The stepwise increase in KL-6 levels observed from dust-exposed workers to simple and complicated silicosis suggests that KL-6 may reflect a continuous disease process rather than a static disease state. Based on this rationale, prospective longitudinal studies are warranted to determine whether these biomarkers can identify individuals at higher risk of progression to complicated silicosis at an earlier stage.

Several limitations should be acknowledged. As a single-center, cross-sectional analysis with a limited sample size, generalizability may be constrained, and the lack of longitudinal follow-up data precludes evaluation of prognostic utility. In addition, potential confounding factors may still exist. Although baseline comparisons across groups showed no statistically significant differences in smoking status (*P* = 0.057) and dust exposure duration (*P* = 0.060), we did not perform multivariable adjustment to fully account for potential residual confounding. Patients with comorbidities other than silicosis were excluded from the final cohort to minimize bias; however, age-related confounding was not adjusted for and may have influenced the observed associations. Moreover, because the present work focused primarily on silicosis, future studies should incorporate additional clinical parameters, expand the range of pneumoconiosis subtypes, and adopt a multi-center design to enhance generalizability. Nevertheless, the strong correlations between KL-6 and multiple immune and inflammatory markers provide a solid foundation for future research. By stratifying patients into three clinical subtypes, we systematically evaluated KL-6 expression patterns and diagnostic values across the silicosis spectrum.

## Conclusion

In this study, we identified a strong correlation between KL-6 and ESR/LDH and demonstrated that their combination significantly improved the early diagnostic accuracy for CS. These findings offer a novel, non-invasive biomarker strategy for early disease detection and monitoring in high-risk occupational populations, with promising implications for real-world clinical applications. In future studies, we will further explore the role of KL-6 in predicting the prognosis of various types of pneumoconiosis and assess its potential as a biomarker for evaluating the therapeutic efficacy of anti-fibrotic treatments in silicosis. Through these investigations, we aim to establish a more convenient tool for diagnosing and prognosticating treatment outcomes in patients with different forms of silicosis.

## Data Availability

The datasets used and/or analyzed during the current study are available from the corresponding author on reasonable request.

## Abbreviations

AUC: Area under the curve
CK: Creatine kinase
CS: Complicated silicosis
DEWs: Dust-exposed workers without silicosis
DLCO: Diffusing capacity of the lung for carbon monoxide
ESR: Erythrocyte sedimentation rate
FEV1: Forced expiratory volume in 1 s
FVC: Forced vital capacity
HRCT: High-resolution computed tomography
IL: Interleukin
KL-6: Krebs von den Lungen-6
LDH: Lactate dehydrogenase
LYM: Lymphocyte count
NEUT: Neutrophil count
NT-pro BNP: N-terminal pro-B-type natriuretic peptide
PFT: Pulmonary function test
PMF: Progressive massive fibrosis
ROC: Receiver operating characteristic
SS: Simple silicosis
6MWT: Six-minute walk test
